# P2X2 Receptor Expression and Function Is Upregulated in the Rat Supraoptic Nucleus Stimulated Through Refeeding After Fasting

**DOI:** 10.3389/fncel.2019.00284

**Published:** 2019-06-26

**Authors:** Milorad Ivetic, Anirban Bhattacharyya, Hana Zemkova

**Affiliations:** Department of Cellular and Molecular Neuroendocrinology, Institute of Physiology, Academy of Sciences of the Czech Republic, Prague, Czechia

**Keywords:** supraoptic nucleus, P2X receptor, P2Y receptor, ATP, GABA, arginine vasopressin, oxytocin

## Abstract

Magnocellular neurons in the supraoptic nucleus (SON), which synthesize and release arginine vasopressin (AVP) and oxytocin (OT), express several subtypes of ATP-stimulated purinergic P2X receptors (P2XR) that modulate neuronal activity as well as neurotransmitter and hormone release. However, the physiological impact of this modulation is not well understood. Here, we tested a hypothesis that P2XRs play a role in the sustained release of hormones from SON neurons stimulated through fasting/refeeding. We studied the effect of 2 h of refeeding after 48 h of fasting on P2XR and P2YR mRNA expression and ATP-induced presynaptic and postsynaptic responses in the SON of 30-day-old rats. Quantitative real-time PCR revealed that the expression of P2X2R and AVP mRNA was upregulated, whereas P2X4R, P2X7R, P2Y2R, and OT mRNA levels were not significantly changed and P2Y1R mRNA expression was decreased. Whole-cell patch clamp recordings performed on isolated rat brain slices showed that the amplitude of the ATP-stimulated somatic current and the ATP-induced increases in the frequency of spontaneous GABAergic inhibitory postsynaptic currents were significantly higher in SON neurons from fasted/refed rats than in SON neurons from normally fed rats. No evidence was found for changes in the presynaptic effect of ATP in SON neurons not expressing somatic P2XRs. These results suggest that the increased activity of SON neurons synthesizing AVP is associated with enhanced expression of P2X2Rs on neuronal cell bodies and their GABAergic presynaptic nerve terminals.

## Introduction

Mammalian magnocellular neurons in the supraoptic nucleus (SON) and paraventricular nucleus (PVN) secrete either vasopressin (AVP) or oxytocin (OT) from their nerve terminals in the posterior pituitary in response to a variety of different physiological stimuli. AVP release increases as a function of plasma osmolality and water status of the body (Oliet and Bourque, 1993; Israel et al., 2010; Choe et al., 2015), whereas OT release is mainly caused by parturition and suckling-induced reflex milk ejection (Moos, 1995; Voisin et al., 1995; Jourdain et al., 1998; Brussaard and Kits, 1999), These two peptides are also locally released in various brain regions (Richard et al., 1991), including within the PVN and SON, where AVP and OT have local regulatory actions (Ludwig, 1998; Kombian et al., 2002; Leng and Ludwig, 2008). The secretion of both hormones is dependent on electrical activity of magnocellular neurons (Leng et al., 1999; Sladek and Kapoor, 2001; Armstrong, 2007; Li et al., 2007; Choe et al., 2015), which is under the control of various excitatory and inhibitory synaptic inputs (Hu and Bourque, 1991; Wuarin and Dudek, 1993; Shibuya et al., 2000; Israel et al., 2010). These inputs, in particular those using glutamate (Decavel and Curras, 1997; Boudaba et al., 2003; Iremonger et al., 2010; Vilhena-Franco et al., 2018) and γ-aminobutyric acid (GABA) (Brussaard and Kits, 1999) as neurotransmitters, play an important role in the generation of the specific pattern of activity in AVP and OT neurons (Moos, 1995; Voisin et al., 1995; Jourdain et al., 1998; Brussaard and Kits, 1999; Israel et al., 2010; Choe et al., 2015).

Two experimental conditions that have been widely used to stimulate the synthesis and release of these peptides are lactation for OT (de Kock et al., 2004; Panatier and Oliet, 2006) and water deprivation or refeeding after fasting for AVP (Carreno et al., 2011; Gottlieb et al., 2011; Yao et al., 2012; Yoshimura et al., 2013; Lucio-Oliveira et al., 2015). Refeeding after fasting represents a complex stimulation to hormone secretion involving the volume/baroreceptors and peripheral/central osmoreceptors (Burlet et al., 1992) that may involve neurotransmitter signaling at different levels. For example, the NR1 subunit of NMDA receptors, expressed in both AVP and OT magnocellular neurons of the SON and PVN, is upregulated after dehydration in these areas (Decavel and Curras, 1997). A higher incidence of GABA(A) receptor-mediated spontaneous inhibitory postsynaptic currents (sIPSCs) has been found in the SON neurons of pregnant rats than in those of virgin rats, consistent with the observations of an increase in the density of GABA-containing synaptic boutons (Brussaard and Kits, 1999).

The secretion of hormones by magnocellular neurosecretory cells in the hypothalamic SON and PVN can be modulated by ATP (adenosine-5′-triphosphate), which is co-released with noradrenaline during stimulation of these cells (Day et al., 1993; Knott et al., 2008; Gordon et al., 2009). Extracellular ATP and its metabolic products, ADP and adenosine, act as agonists or extracellular messengers on ionotropic P2X receptors (P2XRs) and G-protein coupled adenosine receptors and P2Y receptors (P2YRs) (Burnstock, 2006). Locally applied ATP increases cytosolic free Ca^2+^ concentrations in identified somata of dissociated SON neurons (Troadec et al., 1998; Shibuya et al., 1999; Song et al., 2007) and evokes AVP release from isolated posterior pituitary nerve terminals but not OT release (Troadec et al., 1998; Song and Sladek, 2006; Knott et al., 2008; Gomes et al., 2009; Custer et al., 2012; Lemos et al., 2018). In addition, activation of presynaptic P2XRs by the application of ATP dramatically facilitates glutamate and GABA release in SON slices (Vavra et al., 2011).

Although presynaptic P2XR responses have been described in many parts of the brain (Khakh and North, 2012), a satisfactory understanding and the precise physiological function of this form of modulation of synaptic transmission is still lacking. In the present study, we tested a hypothesis that P2XRs might play a role in the release of AVP from SON neurons stimulated through fasting/refeeding. We examined the effect of 2 h of food intake after 48 h of starvation (Lucio-Oliveira et al., 2015) on AVP, OT, P2XR and P2YR mRNA expression and ATP-induced electrophysiological responses in the SON neurons in rat brain slices. Normally fed rats of the same age and sex were used as controls for this condition. We show that fasting/refeeding increases the level of P2X2R mRNA expression and potentiates both postsynaptic and presynaptic ATP-induced responses.

## Materials and Methods

### Animals and Brain Slices

**FIGURE 1:**
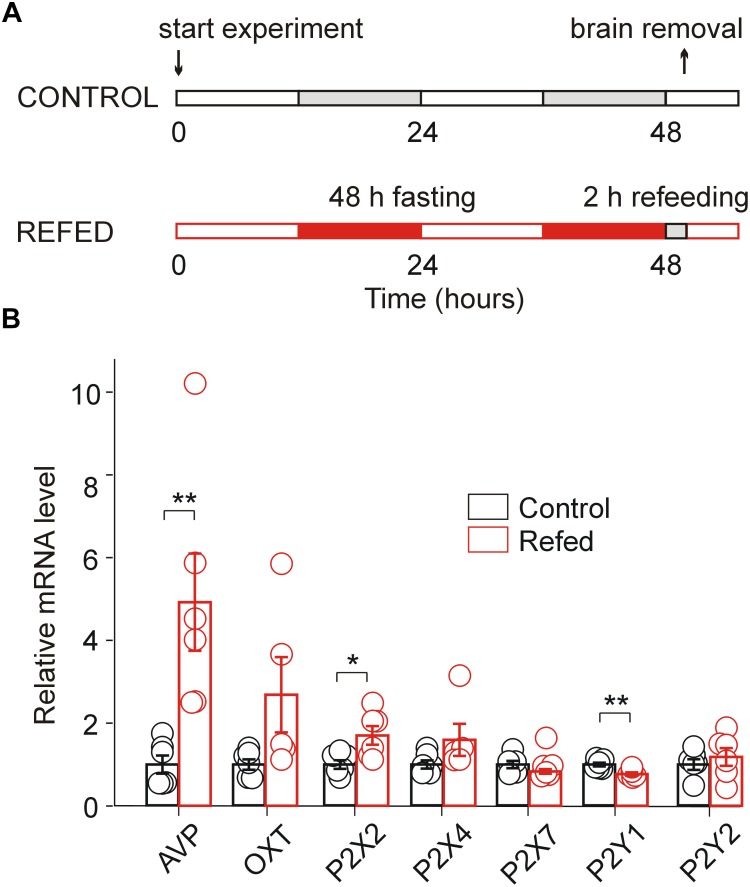
Effect of fasting/refeeding on purinergic P2 receptor expression. **(A)** Schematic diagram of the fasting/refeeding experiment. One group of 30-day-old rats was fed food *ad libitum* for the entire experiment (Control), and the second group of 30-day-old rats was fasted for 48 h and subsequently refed with standard chow for 2 h (Refed). Both groups were allowed to drink water *ad libitum*. At the end of this procedure, hypothalamic slices were prepared from both groups. **(B)** Effect of fasting/refeeding on the relative mRNA expression (calculated as probe/18sRNA ratio) of the P2X2, P2X4, P2X7, P2Y1, and P2Y2 receptors as well as AVP and OT in the SON normalized to the control group. Data represent the mean ± S.E.M with a scatterplot of the individual data points (*n* = 6 tissue samples from three animals). Significant differences between control and fasted/refed animals were estimated using the unpaired *t*-test, *p* < 0.01 (^∗∗^) and *p* < 0.05 (^∗^).

Animals were obtained from established breeding couples in the animal facility (Animal facility of the Institute of Physiology, Czech Academy of Sciences; approval number #56379/2015-MZE-17214). All animal procedures were approved by the Animal Care and Use Committee of the Czech Academy of Sciences (dissection protocol # 67985823). The animals were kept since their birth under conditions of stable temperature and humidity, 12 h light/dark cycles and food and water provided *ad libitum* (Animal Facility of the Institute of Physiology, Czech Academy of Sciences). We employed a previously reported protocol to challenge the peptide producing system of the SON, refeeding with standard chow for 2 h after 2 days of fasting (Figure 1A), which increases the activity of AVP neurons (Lucio-Oliveira et al., 2015). Experiments were performed in 30- to 32-day-old Wistar rats. A total of 64 Wistar rats (37 normally fed and 27 fasted/refed) of both sexes were investigated. Euthanasia was performed by decapitation after anesthesia with isofuranum (Forane, AbbVie s.r.o. Czechia) that does not smell and does not damage epithelium in the respiratory system. Brains were removed and placed into ice-cold (4°C) oxygenated (95% O_2_ + 5% CO_2_) artificial cerebrospinal fluid (ACSF). Hypothalamic slices (200- to 300-μm-thick) containing SONs were cut with a vibratome (DTK-1000, D.S.K., Dosaka, Japan). The slices were allowed to recover for at least 1 h in oxygenated ACSF at 32–33°C before being transferred into a recording chamber. During the experiments, slices were submerged in continuously flowing oxygenated ACSF at 1–2 ml min^−1^ at room temperature (20–25°C). Slices were viewed with an upright microscope (Olympus BX50WI, Melville, NY, United States) mounted on a Gibraltar X-Y table (Burleigh) using a water immersion lens (60× and 10×) and Dodt infrared gradient contrast (Luigs & Neumann, GmbH, Germany). The SON of rats was identified by the position relative to the chiasma opticum (Vavra et al., 2011).

### Quantitative Real-Time RT-PCR

Coronal hypothalamic slices (1000 μm) containing SON(s) were dissected from rat brains using a Vibratome slicer, and SON(s) were punched out of the slices under visual control (magnification 20×) using a needle punch with an internal diameter of approximately 1 mm. Samples were either used immediately or frozen in RNA*later*^TM^ (Sigma-Aldrich) at −80°C. Total RNA was extracted from the tissues using the mirVana^TM^ miRNA Isolation Kit (Ambion^TM^, Thermo Fisher Scientific, United States). Briefly, samples were homogenized in lysis buffer provided with the kit using ceramic balls (MagNA Lyser Green Beads) and a MagNA Lyser homogenizer (Roche Diagnostics GmbH, Germany). RNA was then extracted from the tissue homogenate using acid-phenol:chloroform extraction and subsequent column purification with glass-fiber filter containing columns according to the manufacturer’s protocol. The concentration of total RNA in each sample was measured using a NanoDrop^TM^ spectrophotometer (Thermo Fisher Scientific, Wilmington, DE, United States). Samples were volume adjusted with DNAase and RNAase free DEPC treated water provided with the RT kit and normalized for their RNA content. The first strand cDNA was synthesized from up to 1 μg of isolated RNA using SuperScript^TM^ VILO^TM^ cDNA Synthesis Kit (Invitrogen, Thermo Fisher Scientific, United States) in a 20-μL reaction volume using random primers provided with the kit according to the manufacturer’s protocol. The expression levels of specific mRNA(s) for the AVP, OT, P2X receptors 2, 4, and 7 (P2X2, P2X4, and P2X7), P2Y1 and P2Y2 genes were measured using a ViiA^TM^ 7 Real-Time PCR System (Applied Biosystems, Foster City, United States). The probes and primers (TaqMan^®^ probes) used for these experiments were developed as TaqMan Gene Expression Assay by Applied Biosystems. Specifically, AVP (Rn00566449_m1), OT (Rn00564446_g1), P2X2 (Rn00586491_m1), P2X4 (Rn00580949_m1), P2X7 (Rn00570451_m1), P2Y1 (Rn00562996_m1), and P2Y2 (Rn00568476_m1) were used. Eukaryotic 18 s rRNA endogenous control (VIC^®^/MGB Probe, Primer Limited; Hs99999901_s1) was used as a housekeeping gene/endogenous control. Real-time PCR amplification was performed in 30 μl aliquots on a 96-well fast optical plate in a duplex reaction format. Each reaction contained TaqMan target gene probes labeled with FAM/TAMRA, 18 s RNA probes (VIC/MGB), TaqMan Universal Master Mix II, no UNG (Applied Biosystems) and cDNA. The efficiency of different probes was found to be very similar, and therefore, the 2^−ΔΔ^C_T_ method was used to calculate the relative mRNA levels of all genes of interest normalized to the endogenous control (18 s RNA). Final results were expressed as fold changes in relative mRNA expression with the treatment of the animals (controls vs. 2 h fasted/refed groups).

### Patch Clamp Recordings

ATP-induced currents and membrane potentials were recorded from SON slices using standard whole-cell patch clamp techniques with an Axopatch-200B amplifier (Axon Instruments, Union City, CA, United States). Patch pipettes were pulled on the horizontal Flaming Brown P-97 model puller (Sutter Instruments, Novato, CA, United States) from borosilicate glass (World Precision Instruments, Sarasota, FL) and polished by heat to a tip resistance of 4–6 MΩ. The access resistance (average 14.2 ± 1.2 MΩ, *n* = 18) was monitored throughout each experiment. The mean capacitance of the cells was 6–8 pF, 50–80% series resistance compensation was used, and liquid junction potential (∼4 mV), calculated using the program CLAMPEX 9, was corrected offline when determining the resting membrane potential of SON cells. Data were captured and stored using the pClamp 9 software package in conjunction with the Digidata 1322A A/D converter (Axon Instruments). Signals were filtered at 1 kHz and sampled at 10 kHz. The ATP-induced currents and spontaneous miniature postsynaptic currents (mPSCs) were recorded from cells voltage-clamped in the presence of 0.5 μM tetrodotoxin (TTX) that was used to block action potentials. The cell membrane potential was held at –60 mV.

### Drug Application

ATP (100 μM) was applied in HEPES-buffered extracellular solution (see *Solutions*). The solutions were delivered to the recorded cells by a gravity-driven microperfusion system containing nine glass tubes with a common outlet approximately 300 μM in diameter (RSC-200 Rapid Solution Changer, Biologic, Claix, France). The application tip was routinely positioned approximately 500 μm away from the recorded cell and ∼50 μm above the surface of the slice. The time of each ATP application (5–20 s) was controlled.

### Solutions

Slices were preincubated at 32–33°C in oxygenated ACSF that contained (in mM): 130 NaCl, 3 KCl, 1 MgCl_2_, 2 CaCl_2_, 19 NaHCO_3_, 1.25 NaH_2_PO_4_ and 10 glucose (pH 7.3–7.4; osmolality 300–315 mOsm). ATP was diluted and applied in a N-2-hydroxyethylpiperazine-N′-2-ethanesulfonic acid (HEPES)-buffered extracellular solution (ECS) containing (in mM): 142 NaCl, 3 KCl, 1 MgCl_2_, 2 CaCl_2_, 10 glucose and 10 HEPES, the osmolality was 300–315 mOsm and the pH was adjusted to 7.3 with 1 M NaOH. While testing the effect of ATP application, neurons were exposed to HEPES-buffered ECS for no more than 2 min. The patch electrodes used for whole-cell recording were filled with an intracellular solution containing (in mM): 140 KCl, 3 MgCl_2_, 0.5 CaCl_2_, 10 HEPES and 5 EGTA, and the pH was adjusted to 7.2 with KOH. The osmolality of the intracellular solutions was 285–295 mOsm.

### Data Analysis

The frequencies and amplitudes of miniature GABAergic inhibitory postsynaptic currents (mIPSCs) were manually analyzed off-line using pClamp 10 software (Molecular Devices, United States). The currents were detected using a threshold-based event search and visually evaluated by the experimenters. Only events exceeding 10 pA and lasting 15–20 ms were used in subsequent analysis. Miniature glutamatergic excitatory postsynaptic currents (mEPSCs) identified as smaller and shorter (∼5 ms) events (Vavra et al., 2011), were ignored. The amplitudes of individual events were determined by the detection program, and the frequency was calculated by dividing number of events by the recording time. The average time of recordings used for frequency calculation was 19.6 ± 0.4 s and 8.6 ± 1.0 s (*n* = 10) before and after ATP application, respectively. The kinetics of mEPSC and mIPSC current decay were fitted by a single exponential function [*y* = A exp(−t/τ)] and by the sum of two exponentials [*y* = A1 exp(−t/τ_1_) + A2 exp(−t/τ_2_)], respectively, using the program CLAMPFIT 9, where A1 and A2 are relative amplitudes of the first and second exponential, and τ_1_ and τ_2_ are time constants. The derived time constant for current decay was labeled as τ. All values are reported as the mean ± SEM. Significant differences were determined by two-way analysis of variance (ANOVA) and Tukey’s *post hoc* test using SigmaStat 2000 v9.01, or Student’s *t*-test using SigmaPlot v10.01 with *p* < 0.01 (^∗∗^) and *p* < 0.05 (^∗^). Graphing was performed using SigmaPlot and CorelDraw software.

### Chemicals

TTX was from Tocris Bioscience. ATP and all other chemicals were from Sigma (St. Louis, MO).

## Results

### Upregulation of AVP and P2X2R mRNA in the Hypothalamic SON Stimulated Through Fasting/Refeeding

Our previous analysis of the mRNA expression of seven P2XRs (P2X1–P2X7) and three P2YRs (P2Y1, P2Y2, and P2Y12) in rat SON tissue revealed significant differential expression (P2X2R > P2X7R > P2X4R > P2Y1R = P2Y2R), while the mRNA expression for the other P2 receptors was minimal (Vavra et al., 2011). Therefore, to address whether the increased synthesizing and releasing activity of AVP neurons induced by the fasting/refeeding protocol (Figure 1A; Lucio-Oliveira et al., 2015) regulates P2XR gene expression in the SON, we performed quantitative PCR analysis with reverse transcription (RT-qPCR) to compare the mRNA expression of P2X2R, P2X4R, P2X7R, P2Y1R, and P2Y2R in SON tissue from 30-day-old rats refed for 2 h after 48 h of starvation with that in SON tissue isolated from controls, i.e., normally fed rats (Figure 1B). Additionally, we examined the mRNA expression levels of AVP and OT in both animal groups (Figure 1B). These experiments showed a significant increase in the expression of AVP (ratio = 4.93 ± 1.17, *n* = 3, unpaired *t*-test, *p* = 0.0074) and P2X2R (ratio = 1.61 ± 0.17, *n* = 3, *p* = 0.0199) mRNA, a decrease in P2Y1 mRNA (ratio = 0.78 ± 0.04, *n* = 3, *p* = 0.0060) in fasted/refed animals compared to controls (Figure 1B). However, changes in the mRNA expression of OT, P2X4R, P2X7R, and P2Y2R were non-significant. These results showed that fasting/refeeding increases the expression of P2X2R and this effect correlates with the upregulation of AVP in the SON.

### Biophysical Properties of SON Neurons in Slices From Fasted/Refed Rats and Controls

**FIGURE 2:**
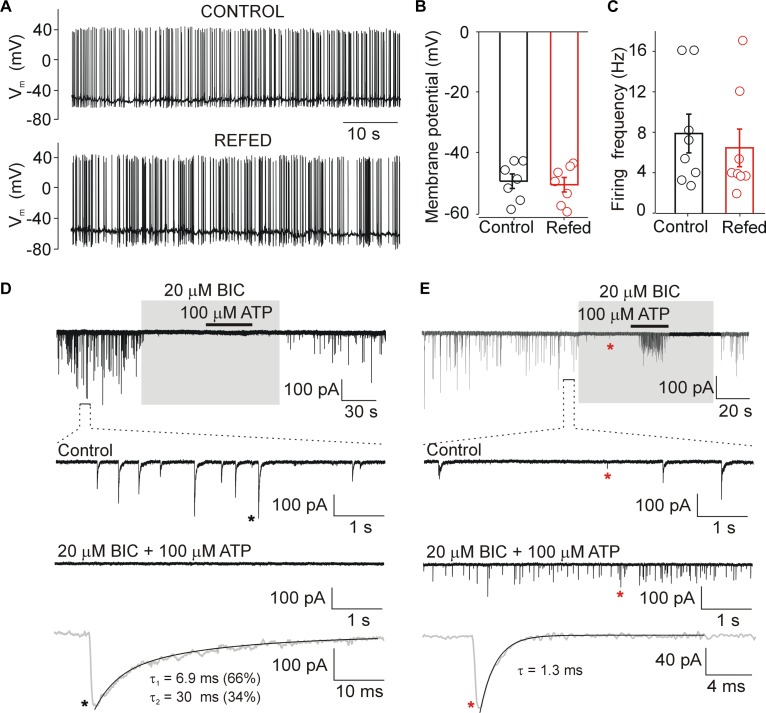
Biophysical properties of SON neurons. **(A)** Action potentials recorded from SON neurons in slices from normally fed (Control) and fasted/refed (Refed) animals using current clamp patch clamp configuration. Resting membrane potential **(B)** and frequency of action potentials **(C)** in both animal groups. Data represent the mean ± S.E.M of 6–8 cells from three independent experiments. **(D,E)** Miniature postsynaptic currents recorded from SON neurons voltage-clamped at –60 mV in the presence of TTX (0.5 μM). (D) Example record from cell with mIPSCs. Application of bicuculline (BIC, 20 μM) inhibited all GABAergic synaptic currents both in the presence and absence of ATP (100 μM). Lower trace shows mIPSC (black asterisk) on expanded time scale. **(E)** Example record from cell exhibiting both mIPSCs and mEPSCs. Application of bicuculline (BIC, 20 μM) inhibited GABAergic synaptic currents and glutamatergic excitatory postsynaptic currents persisted. Lower trace shows mEPSC (red asterisk, in the presence of ATP and BIC) on expanded time scale. The time constants (τ, τ_1_, and τ_2_) were measured by fitting the curve to a monoexponential (mEPSCs.) or biexponential (mIPSCs) functions.

We used acutely isolated hypothalamic slices to determine the basic electrophysiological properties of SON neurons from fasted/refed and normally fed rats. Whole-cell current clamp recordings performed 1–6 h after isolation showed that the resting membrane potential of SON neurons was −49.1 ± 2.3 mV (*n* = 8 cells) in control rats and −50.7 ± 2.3 mV (*n* = 8 cells) in fasted/refed rats (Figure 2A,B). The average frequency of action potentials was 6.5 ± 1.8 mV (*n* = 8) and 7.9 ± 1.9 mV (*n* = 8, *p* = 0,571) in control and fasted/refed rats, respectively (Figure 2A,C). Thus, no significant change in the basal membrane properties and spontaneous neuronal activity was observed in SON neurons stimulated to secrete hormones through fasting/refeeding.

In the presence of 0.5 μM TTX, whole-cell voltage clamp recordings showed that under our experimental conditions (intracellular [Cl^−^], 144 mM; extracellular [Cl^−^], 151 mM; holding potential, −60 mV; the theoretical equilibrium potential for Cl calculated by the Nernst equation is about 0 mV), both mEPSCs and mIPSCs could be recorded as small inward currents (Figure 2D,E). Using ATP to stimulate the frequency of mEPSCs and mIPSCs (Vavra et al., 2011) and bicuculline (BIC; 20 μM) to block GABAergic currents, we found that mEPSCs and mIPSCs differ in their amplitudes and decay time constants. The amplitude of BIC-sensitive currents (mIPSCs) was 72 ± 4 pA, and the decay phase was well fitted by a sum of two exponentials with weight time constant of 6.1 ± 0.6 ms (contribution 41 ± 16%) and 33 ± 6 ms (*n* = 8 cells; Figure 2D). The amplitude of BIC-insensitive currents was much smaller, 22 ± 4 pA, and the decay phase was well fitted with a single exponential with a time constant of 1.5 ± 0.3 ms (*n* = 5 cells; Figure 2E). Previously we have shown that these events are inhibited with 6,7-dinitroquinoxaline-2, 3-dione (DNQX; 20 μM) that blocks α-amino-3-hydroxy-5-methyl-4-isoxazolepropionic acid (AMPA) currents and 2-amino-5-phosphonopentanoic acid (AP5; 50 μM) that blocks *N*-Methyl-D-aspartate (NMDA) currents (Vavra et al., 2011), indicating that these are mEPSCs. These events were observed in only about 30% of SON neurons from both animal groups, and were not studied further. The differences in the amplitude and time course allowed us to examine the effects of ATP on mIPSCs in the absence of specific glutamatergic blockers (see Methods for mEPCs discrimination). The GABAergic mIPSCs could be recorded in all SON neurons, and the average basal frequency was not significantly different between the two animal groups (control, 2.9 ± 0.3 Hz, *n* = 60; fasted/refed, 2.2 ± 0.2 Hz; *n* = 52; *p* = 0.5745). The frequency of mEPSCs was 0.24 ± 0.10 Hz (*n* = 6) and 0.32 ± 0.08 Hz (*n* = 8; *p* = 0,4556) in controls and fasted/refed rats, respectively.

### Increased Amplitude of ATP-Induced Somatic Current in the SON Neurons of Fasted/Refed Rats

**FIGURE 3:**
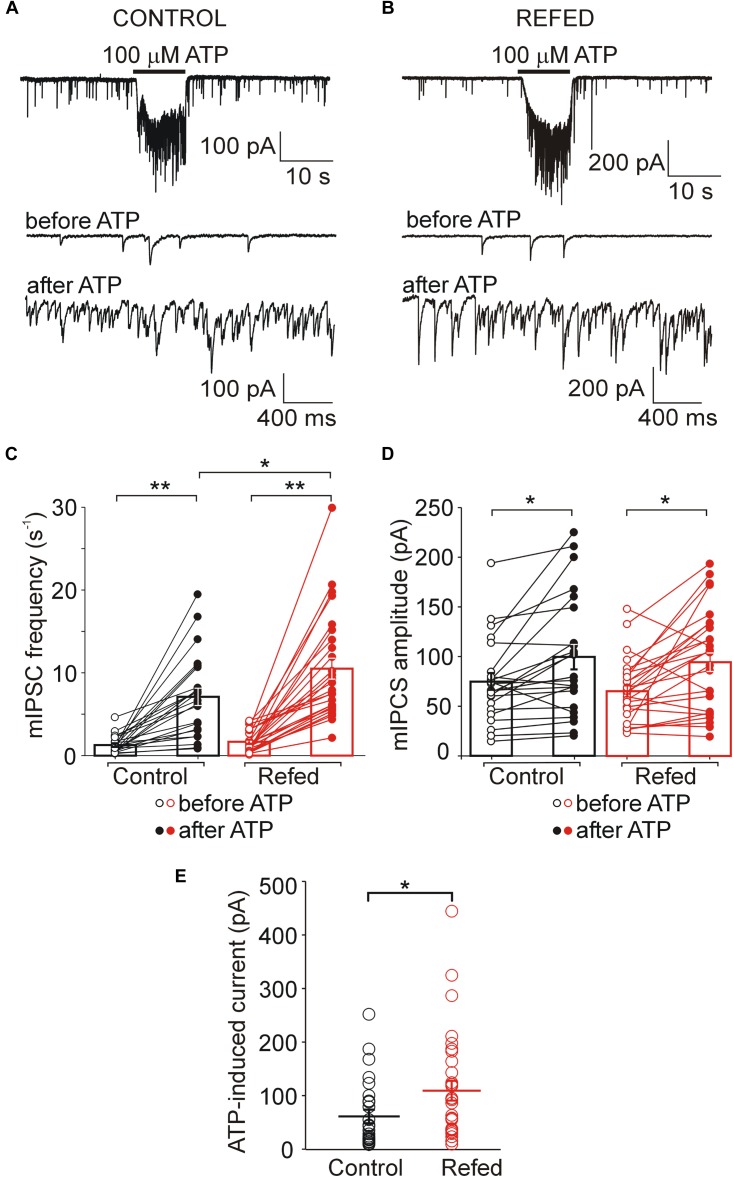
The difference between control and fasted/refed rats in ATP-induced somatic and presynaptic effects. ATP evoked somatic current and increased the frequency of mIPSC in SON neurons from control **(A)** and fasted/refed **(B)** animals. Traces on an expanded time scale show spontaneous inhibitory synaptic currents before and after ATP application. **(C,D)** The frequency **(C)** and amplitude **(D)** of mIPSCs in control (black) and refed (red) animals in the presence (closed symbols) and absence (open symbols) of ATP. Data represent the mean ± S.E.M with a scatterplot of the individual data points (control, *n* = 17 cells; refed, *n* = 26 cells). **(E)** The amplitude of current induced by 5–10 s ATP application. Data represent the mean ± S.E.M with a scatterplot of the individual data points (control, *n* = 26 cells; refed, *n* = 34 cells). Analysis was performed by two-way ANOVA and Tukey’s *post hoc* test. ^∗∗^*p* < 0.01, ^∗^*p* < 0.05. The ATP-induced current and ATP-induced frequency increase were significantly higher in fasted/refed rats than control rats.

**FIGURE 4:**
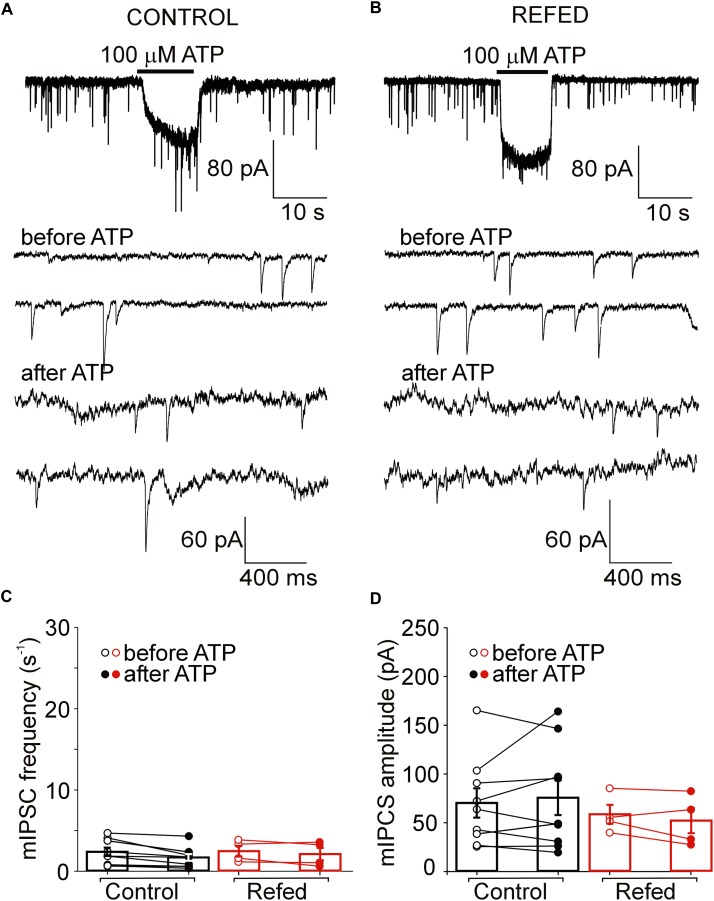
The absence of a presynaptic effect of ATP in a subpopulation of P2XR-expressing SON neurons in both animal groups. **(A,B)** ATP-induced current without increases in the frequency of mIPSCs in control **(A)** and refed **(B)** animals. Traces on an expanded time scale show spontaneous inhibitory synaptic currents before and after ATP application **(C,D)** The frequency **(C)** and amplitude **(D)** of mIPSCs in control (black) and refed (red) animals in the presence (closed symbols) and absence (open symbols) of ATP. Data represent the mean ± S.E.M with a scatterplot of the individual data points (control, *n* = 9 cells; refed, *n* = 4 cells).

Next, we applied 100 μM ATP to SON neurons voltage-clamped at −60 mV to characterize ATP-induced somatic current. This concentration of ATP is close to the effective concentration producing a half-maximal effect of ATP in SON slices from control rats [EC_50_ = 70 ± 10 μM; (Vavra et al., 2011)], and was used throughout the study. Voltage clamp whole cell recording revealed that the brief application (5-10 s) of ATP evoked inward currents in 26 out of 60 control neurons (57 slices from 37 rats; Figure 3A, 4A) and in 30 out of 52 neurons from fasted/refed rats (43 slices from 27 rats; Figure 3B, 4B). The mean amplitude of ATP-induced somatic current was significantly higher in fasted/refed rats than controls (control, 75 ± 18 pA; fasted/refed, 132 ± 25 pA; *p* = 0.043; Figure 3E). These results indicate that refeeding after fasting upregulates the P2XR protein level on the cell somata of SON neurons compared to SON neurons from normally fed rats.

### Increased ATP-Induced Potentiation of GABA Release in the P2XR-Expressing SON Neurons of Fasted/Refed Rats

In addition to the amplitude of ATP-evoked somatic current, we studied the effect of presynaptic P2XR activation on the frequency and amplitude of miniature mIPSCs. We first examined the effect of ATP on mIPSC frequency in SON neurons exhibiting ATP-evoked somatic current (Figure 3, 4); these neurons will be called “P2XR-expressing” neurons in this study. In 65% (17/26) of control P2XR-expressing SON neurons, the application of ATP increased the mIPSC frequency by 1198 ± 202% (before ATP, 1.29 ± 0.23 Hz; after ATP, 8.60 ± 1.26 Hz; *p* = 0.00005; Figure 3C, left) and increased the amplitude from 66.60 ± 7.38 pA to 99.71 ± 15.33 pA (*p* = 0.0055; Figure 3D, left). The remaining 35% (9/26) of cells showed ATP-evoked somatic current without ATP-induced increase in mIPSC frequency (before ATP, 2.35 ± 0.52 Hz; after ATP, 1.95 ± 0.42, Figure 4C, left) and amplitude (before ATP, 68.98 ± 15.01 pA; after ATP, 76.30 ± 17.78 pA; *n* = 9, Figure 4D, left).

In fasted/refed rats, ATP-induced increase in mIPSC frequency and amplitude was observed more often, increasing to 87% (26/30) of P2XR-expressing neurons (Figure 3B). The application of ATP increased the mIPSC frequency by 1102 ± 241% (before ATP, 1.45 ± 0.23; after ATP, 10.79 ± 1.35 Hz; *p* = 0.0000005; Figure 3C, right) and increased the amplitude from 64.01 ± 5.35 pA to 98.16 ± 9.81 pA (*p* = 0.0003, Figure 3D, right). The difference between control and fasted/refed rats in the ATP-induced increase in mIPSC frequency was significant (two-way ANOVA, *p* = 0.0469; Figure 3C), but the difference in the ATP-induced increase in mIPSC amplitude was not (Figure 3D). The remaining 13% (4/30) of cells from fasted/refed animals exhibited ATP-evoked somatic current without ATP-induced increase in mIPSC frequency (before ATP, 2.25 ± 0.85 Hz; after ATP, 2.07 ± 0.75 Hz; Figure 4C, right) and amplitude (before ATP, 58.94 ± 9.67 pA; after ATP, 53.46 ± 10.15 pA; *n* = 4, Figure 4D, right).

These data revealed that fasting/refeeding increases expression of the P2XRs in the nerve terminals releasing GABA on P2XR-expressing SON neurons.

### No Effect of Refeeding After Fasting on ATP-Evoked Presynaptic Responses in a Subpopulation of SON Neurons Not Expressing Somatic P2XRs

**FIGURE 5:**
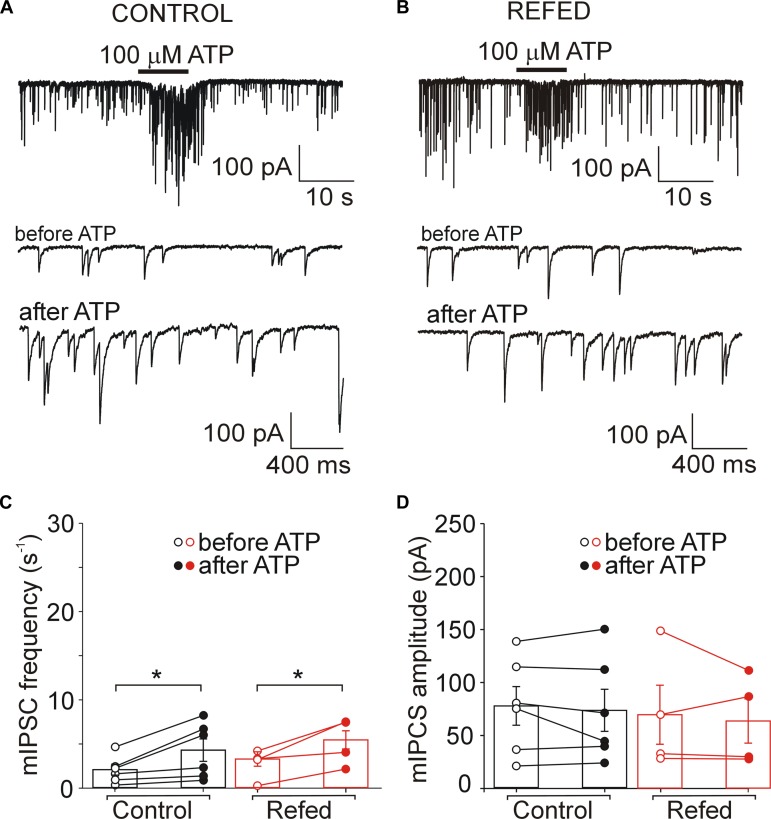
Presynaptic effect of ATP in SON neurons not expressing P2XRs is similar in both animal groups. **(A,B)** ATP-induced increases in the frequency of mIPSCs in control **(A)** and refed **(B)** animals. Traces on an expanded time scale show spontaneous inhibitory synaptic currents before and after ATP application **(C,D)** The frequency **(C)** and amplitude **(D)** of mIPSCs in control (black) and refed (red) animals in the presence (closed symbols) and absence (open symbols) of ATP. Data represent the mean ± S.E.M with a scatterplot of the individual data points (control, *n* = 6 cells; refed, *n* = 4 cells). Analysis was performed by two-way ANOVA and Tukey’s *post hoc* test. ^∗^*p* < 0.05.

**FIGURE 6:**
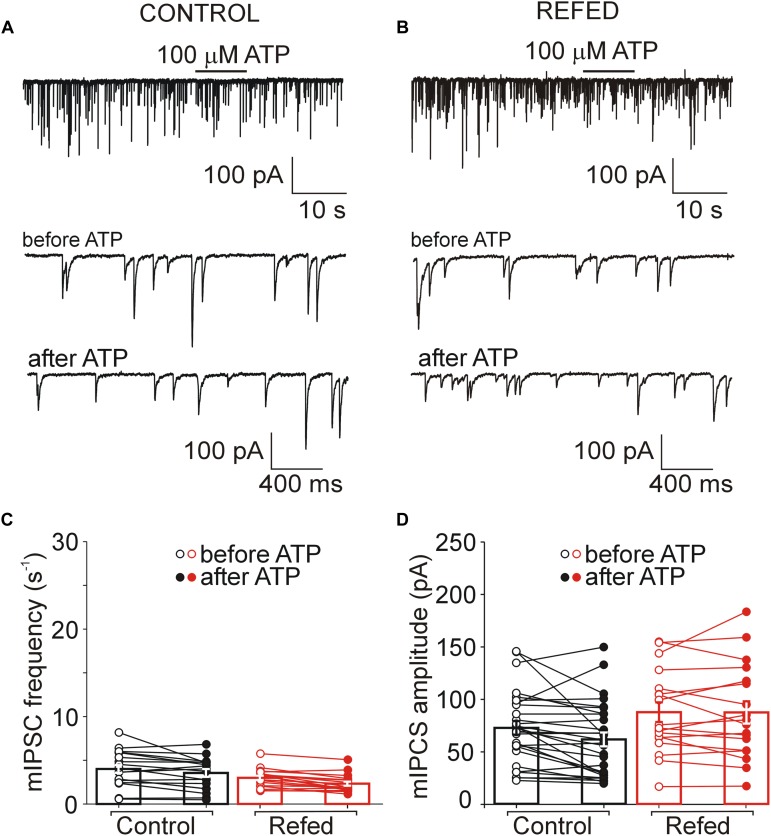
The absence of both somatic and presynaptic effects of ATP in a subpopulation of SON neurons. **(A,B)** Lack of effect of ATP on the frequency of mIPSCs in control **(A)** and refed **(B)** animals. Traces on an expanded time scale show spontaneous inhibitory synaptic currents before and after ATP application **(C,D)** The frequency **(C)** and amplitude **(D)** of mIPSCs in control (black) and refed (red) animals in the presence (closed symbols) and absence (open symbols) of ATP. Data represent the mean ± S.E.M with a scatterplot of the individual data points (control, *n* = 28 cells; refed, *n* = 18 cells).

Next, we investigated the effect of ATP on GABAergic synaptic transmission in SON neurons not exhibiting ATP-evoked somatic current (Figure 5, 6). In 18% (6/34) of control SON neurons, ATP application increased mIPSC frequency by 207 ± 22% (before ATP, 2.06 ± 0.62 Hz; after ATP, 4.27 ± 1.26 Hz; *p* = 0.031; Figure 5A,C, left), without impacting the amplitude (before ATP, 67.30 ± 20.56 pA; after ATP, 63.01 ± 22.51 pA; *p* = 0.509; Figure 5D, left). The remaining 82% (28/34) of neurons exhibited no effect of ATP on mIPSC frequency (before ATP, 4.05 ± 0.44 Hz; after ATP, 3.59 ± 0.45 Hz; Figure 6A,C, left) and amplitude (before ATP, 72.61 ± 6.49 pA; after ATP, 60.78 ± 6.51 pA; *n* = 28; Figure 6D, left).

Similarly, in 18% (4/22) of SON neurons from fasted/refed animals, ATP application increased mIPSC frequency by 296 ± 140% (before ATP, 3.02 ± 1.00 Hz; after ATP, 4.95 ± 1.26 Hz; *p* = 0,048; Figure 5B,C, right), without impacting sIPSC amplitude (before, 69.61 ± 27.87 pA; after ATP, 63.68 ± 20.88 pA; *p* = 0,638; Figure 5D, right). The difference between control and fasted/refed rats in the ATP-induced increase in mIPSC frequency was not significant (two-way ANOVA, *p* = 0.6660). The remaining 82% (18/22) of cells displayed no ATP-induced increase in mIPSC frequency (before ATP, 3.02 ± 0.25 Hz; after ATP, 2.35 ± 0.24 Hz; Figure 6B,C, right) and amplitude (ECS, 85.36 ± 9.61 pA; ATP, 84.97 ± 10.91 pA; *n* = 18; Figure 6D, right).

These data reveal that fasting/refeeding has no effect on presynaptic ATP-induced responses in SON neurons not expressing somatic P2XRs. In addition, these data also show that the amplitude of postsynaptic current did not increase when ATP application induces moderate increase in mIPSC frequency.

## Discussion

We found significantly increased AVP and P2X2R expression and decreased P2Y1R expression, but no changes in OT, P2X4R, P2X7R, and P2Y2R mRNA expression in the rat SON after 48 h of starvation and 2 h after food intake. Using acutely isolated rat brain slices, we showed that the changes in P2X2R mRNA expression are accompanied by functional effects such that the amplitude of ATP-stimulated somatic current and the incidence of P2XRs mediated presynaptic facilitation of GABA release onto P2XR-expressing SON neurons increases. These data suggest that the recruitment of P2X2Rs to both postsynaptic and presynaptic sites could be associated with the increased synthesis and release of AVP in the SON of fasted/refed rats.

Food deprivation for 48 h causes a decline in the AVP level in the SON, while little change in the OT concentration was detected (Burlet et al., 1992). Food intake after 48 h of fasting evokes increases in plasma AVP (Lucio-Oliveira et al., 2015) and OT (Lucio-Oliveira and Franci, 2012) levels. Previous studies also revealed that 2–4 h of refeeding after 48 h of fasting significantly increases the mRNA levels of both OT and AVP in the mouse hypothalamus compared to the OT and AVP mRNA expression in normally fed mice (Poplawski et al., 2010). Refeeding after fasting increases the expression of the immediate early gene c-FOS in identified AVP and, to a lesser extent, OT cells, indicating that AVP-positive neurons in the SON could show higher activity than OT-positive neurons under these experimental conditions (Timofeeva et al., 2005; Johnstone et al., 2006; Kohno et al., 2008; Lucio-Oliveira and Franci, 2012; Lucio-Oliveira et al., 2015). In agreement with this, we found that refeeding after fasting significantly increases AVP mRNA expression, while changes in OT mRNA expression were not significant.

Previous studies on the hypothalamic SON and/or PVN showed the presence of mRNA transcripts not only for P2X2 but also for the P2X3, P2X4, P2X6, and P2X7 receptors (Bo et al., 1995; Collo et al., 1996; Vulchanova et al., 1996; Xiang et al., 1998; Shibuya et al., 1999; Vavra et al., 2011). Experiments with specific P2XR knockout mice revealed that endogenously released ATP acts on P2X2R but not P2X3R or P2X7R in posterior pituitary nerve terminals (Custer et al., 2012). Functional and pharmacological studies on SON neurons also identified P2X2R as a dominant subtype of the P2X receptor (Troadec et al., 1998; Song and Sladek, 2006; Gomes et al., 2009; Vavra et al., 2011). P2X2R activation increases the release of AVP from hypothalamo-neurohypophyseal system explants (Troadec et al., 1998; Song and Sladek, 2006; Gomes et al., 2009) and evokes somatic current in the SON neurons of hypothalamic slices (Vavra et al., 2011; Bhattacharya et al., 2013). There are lines of evidence indicating that P2X2R-expressing neurons are magnocellular AVP neurons. First, locally applied ATP increases cytosolic free Ca^2+^ concentrations in identified somata of dissociated AVP neurons from the SON (Troadec et al., 1998; Shibuya et al., 1999; Song et al., 2007) and evokes AVP release from isolated posterior pituitary nerve terminals, but no significant OT release (Troadec et al., 1998; Song and Sladek, 2006; Gomes et al., 2009; Lemos et al., 2018). Second, ATP endogenously released from the posterior pituitary during electrical stimulation depolarizes the nerve terminals and potentiates AVP secretion (Knott et al., 2008). Finally, the data presented here show that the increased expression of P2X2R mRNA and functionality in terms of ATP-induced current in the SON of 2 h refed animals corresponds with the increased expression of AVP mRNA. Thus, these results altogether support the idea that the upregulation of P2X2Rs during fasting/refeeding could be selectively associated with increased synthesizing and releasing activity of AVP neurons.

P2X2R has also been shown to be expressed on presynaptic nerve terminals in hypothalamic slices and its activation facilitates glutamate and GABA release in a subpopulation of SON neurons (Vavra et al., 2011; Bhattacharya et al., 2013). The ATP-induced increase in mIPSC amplitude observed here in P2XR-expressing neurons might represent multiquantal release of GABA that was due to a dramatic influx of calcium through a high number of presynaptic P2X2R channels. This idea is supported by the fact that the amplitude of postsynaptic current did not increase when ATP-induced increase in frequency was moderate, such as in neurons not-expressing somatic P2XRs. Quantitative analysis of GABA-synthesizing enzyme glutamate decarboxylase or GABA immunostaining combined with OT and AVP immunolocalization previously showed that GABAergic innervation within the SON is very extensive and uniformly distributed within the nucleus and that GABAergic nerve terminals contact OT and AVP neurons to a similar extent (Theodosis et al., 1986; Meeker et al., 1993). Electron microscopy observations has also shown that not all axons in the rat SON display immunoreactivity to P2X2Rs (Loesch et al., 1999). Our electrophysiology results revealed that most of P2XR-expressing GABAergic inputs terminate on P2XR-expressing neurons (probably AVP neurons), while most GABAergic inputs without presynaptic P2XRs terminate on neurons not expressing P2XRs (probably OT neurons). These results also support idea that upregulation of P2X2Rs during fasting/refeeding is selectively associated with increased activity of AVP neurons, in this case at presynaptic level.

Both P2XRs and P2YRs participate in the ATP-induced increase in [Ca^2+^]_i_ in the SON cells (Song et al., 2007) and P2YRs have been reported in rat SON astrocytes (Espallergues et al., 2007). P2YRs couple with the phospholipase C (PLC) pathway, the activation of which results in [Ca^2+^]_i_ increase due to Ca^2+^ release from intracellular stores and the stimulation of a Ca^2+^-dependent K^+^ current (Schicker et al., 2010). This indicates that P2YR activation might hyperpolarize the membrane and inhibit neuronal firing. In this scenario, reduced expression of P2Y1R mRNA might contribute to increased excitatory effect of ATP on neuronal somata. Since ADP was not found to significantly inhibit the frequency of action potentials in SON neurons of slices (Vavra et al., 2011), functional consequences of reduced expression of P2Y1R mRNA observed in SON from fasted/refed animals need further investigation.

SON neurons receive GABAergic afferent inputs from osmosensitive neurons of the circumventricular subfornical organ that communicate chronic changes in plasma osmolality through direct projections to the AVP neurons, thus modulating the synthesis of AVP as well as its transport to and release from the posterior lobe of the pituitary (Weiss and Hatton, 1990). Some inputs to AVP cells could originate from GABA-containing cells in the locus coeruleus (Jones and Moore, 1977; Leng et al., 1999) or interneurons within the perinuclear zone (Tappaz et al., 1983), which presumably mediate the projections from other regions, and regulate the excitatory and inhibitory inputs into the SON neurons (Leng et al., 1999; Wang et al., 2015). Neurons in this zone could account for the large number of intact synapses remaining in the SON after its surgical isolation by slicing (Leranth et al., 1975). The perinuclear GABAergic neurons are thought to mediate the rapid inhibition of AVP neurons following transient hypertension (Jhamandas et al., 1989; Nissen et al., 1993). We can speculate that the observed food-intake related changes in GABAergic synaptic input might be associated with changes in the patterning of the discharge of AVP neurons so that synthesis and release of AVP is increased.

Overall, our observations indicate that ATP and P2X2R signaling could be significantly linked with the complex stimulation of AVP neurons and corresponding hormone secretion induced in rats by refeeding after fasting.

## Data Availability

The raw data supporting the conclusions of this manuscript will be made available by the authors, without undue reservation, to any qualified researcher.

## Ethics Statement

Animals were obtained from established breeding couples in the animal facility (Animal facility of the Institute of Physiology, Czech Academy of Sciences; approval number #56379/2015-MZE-17214). All animal procedures were approved by the Animal Care and Use Committee of the Czech Academy of Sciences (dissection protocol # 67985823).

## Author Contributions

MI prepared rat brain slices, carried out electrophysiological experiments, and contributed to the conception of the work. AB did quantitative real-time RT-PCR. HZ designed the experiments and finalized the manuscript. All authors analyzed the data, contributed to drafting the work, approved the version to be published, and agreed to be accountable for all aspects of the work.

## Conflict of Interest Statement

The authors declare that the research was conducted in the absence of any commercial or financial relationships that could be construed as a potential conflict of interest.
